# Evaluating the effectiveness of a multi-component intervention on early childhood development in paediatric HIV care and treatment programmes: a randomised controlled trial

**DOI:** 10.1186/s12887-018-1201-0

**Published:** 2018-07-09

**Authors:** R. Chingono, H. Mebrahtu, Z. Mupambireyi, V. Simms, H. A. Weiss, P. Ndlovu, F. Charasika, M. Tomlinson, L.D. Cluver, F. M. Cowan, L. Sherr

**Affiliations:** 1 Centre for Sexual Health HIV/AIDS Research (CeSHHAR), Harare, Zimbabwe; 2 Department of Infection and Population Health, University College London, London, UK; 3 MRC Tropical Epidemiology Group, London School of Hygiene and Tropical Medicine, London, UK; 4 World Education Inc./Bantwana (WEI/B), Harare, Zimbabwe; 5 Department of Psychology, Stellenbosch University, Stellenbosch, South Africa; 6 University of Cape Town, Cape Town, South Africa; 7 Department of International Public Health, Liverpool School of Tropical Medicine, Liverpool, UK

**Keywords:** Early childhood stimulation, Internal savings and lending scheme, Case management, HIV exposed infants, Zimbabwe

## Abstract

**Background:**

HIV infection in a family may affect optimum child development. Our hypothesis is that child development outcomes among HIV-exposed infants will be improved through a complex early childhood stimulation (ECS) programme, and income and loans saving programme for HIV positive parents.

**Methods:**

The study was a cluster-randomized controlled trial in 30 clinic sites in two districts in Zimbabwe. Clinics were randomised in a 1:1 allocation ratio to the Child Health Intervention for Development Outcomes (CHIDO) intervention or Ministry of Health standard care. The CHIDO intervention comprises three elements: a group ECS parenting programme, an internal savings and lending scheme (ISALS) and case-management home visits by village health workers. The intervention was aimed at caregiver-child dyads (child aged 0–24 months) where the infant was HIV exposed or infected. The primary outcomes were cognitive development (assessed by the Mullen Scales of Early Learning) and retention of the child in HIV care, at 12 months after enrolment. A comprehensive process evaluation was conducted.

**Discussion:**

The results of this cluster-randomised trial will provide important information regarding the effects of multi-component interventions in mitigating developmental delays in HIV-exposed infants living in resource-limited environments.

**Trial registration:**

This trial is registered with the Pan African Clinical Trials Registry (www.pactr.org), registration number PACTR201701001387209; the trial was registered on 16th January 2017 (retrospectively registered).

## Background

Early childhood experiences shape the long-term physical, emotional, and psychological health in children [1]. Improving these experiences using early childhood stimulation (ECS) could have long-term positive impacts on academic achievements, and socialisation skills [2]. In addition to appropriate ECS, having a consistent, responsive caregiver and a stable environment during these early years contributes to promoting optimal health and development [3, 4].

Child development opportunities are enhanced with good quality caregiving, stimulating environments, adequate nutrition, health care, protection, and socialisation. International literature has shown that parenting interventions can be effective in changing parenting behaviour [5] and subsequently enhancing various child outcomes [6, 7]. Conversely, HIV, especially in the context of poor socioeconomic environments, can threaten child survival and well-being [8]. HIV can affect child development indirectly through factors such as access to quality childcare, food insufficiency, economic hardships, unemployment, and bereavement [9, 10]. Studies on children infected [11] and affected [12] by HIV have shown poorer child development outcomes than uninfected and unaffected children [13], with considerably more evidence on adolescents than young children [14], and greater effect shown in resource-poor settings [15]. Studies in low-and middle-income countries have demonstrated sustainable benefits of interventions aimed at enhancing child development with improvements in cognitive and developmental outcomes [16–19].

HIV affects parenting ability and strategies in a number of ways. Parents living with HIV may be distracted with their own physical and mental health concerns which can potentially affect the quality of care and attention devoted to the child [20–22]. Although the importance of ECS to child development is well established [3, 4] and parenting interventions have been shown to be effective in both resource rich and resource poor settings [23], little has been done to look at the impact of ECS in environments where HIV is generalised and also among very young children.

Many children in Sub-Saharan Africa face severe challenges affecting early development. Southern Africa has a generalised HIV epidemic. The current prevalence of HIV is estimated to be 14.6% among 15–64 year olds in Zimbabwe [24]. Despite notable reductions in the mother-to-child transmission rates, to 6.7%, at the population level [25], UNAIDS estimated that mother-to-child transmission is 6.39% [26]. The number of children orphaned due to AIDS is estimated as 524,581 [26]. High poverty (72% consumption poverty) with 25% of children living in extreme poverty [27] may also be a driver of poor child outcome.

**Fig. 1:**
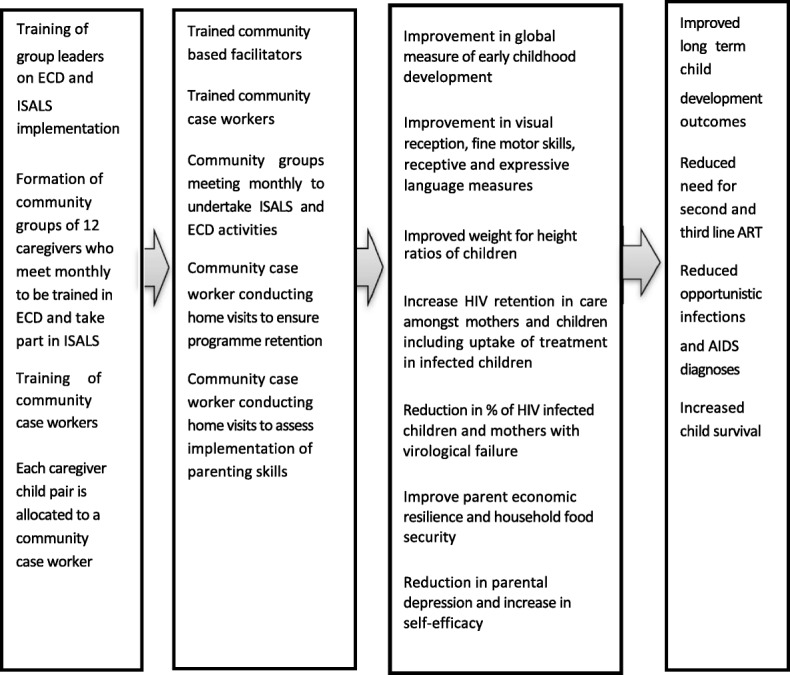
Proposed theory of change

Developmental challenges have been well documented in both HIV positive and HIV exposed infants [28–31]. The mechanisms are unclear. Exposure to the virus, antiretroviral (ART), or living in a family with HIV may all contribute, with the concomitant environmental risks associated with having parent(s) who are HIV-positive being one of the most influential factors among all those included. Effective interventions to mitigate these are available, and when implemented by the caregivers of HIV positive and HIV exposed children, it can improve their performance [12, 32, 33]. The intervention, we hypothesise, will result in improved long-term child development outcomes, reduced need for second and third line ART, reduced opportunistic infections and AIDS diagnoses and increased child survival (Fig. 1).

## Methods

### Aims and objectives

The overall aim of this trial was to determine the impact of a comprehensive community-based intervention set up to simultaneously enhance child stimulation, reduce economic insecurity and improve retention in care among HIV exposed and infected children aged 0–24 month.

The specific aims of the trial include:

1. To pilot an intervention aimed at improving early childhood development (ECD), strengthening household economic resilience, enhancing adherence and retention in paediatric HIV care and treatment programs amongst caregivers and their HIV exposed children aged 0–24 months in Zimbabwe and modify the intervention in line with findings,
2. To evaluate the final intervention using a cluster-randomized controlled trial design,
3. To disseminate findings to influence policy and programming in Zimbabwe.

### Collaborations

This trial was a collaborative project run by researchers from University College London, Centre for Sexual Health and HIV /AIDS Research (CeSHHAR) Zimbabwe, Liverpool School of Tropical Medicine, London School of Hygiene and Tropical Medicine, World Education, Stellenbosch University and Oxford University. CeSHHAR Zimbabwe was responsible for implementing and conducting all the research-oriented activities. World Education was responsible for providing technical support and ensuring delivery of the intervention by their implementing partner, Mavambo Orphan Care, as well collection of routine monitoring and evaluation data.

### Trial setting

The trial was set in 30 health facilities in two districts in rural Zimbabwe, Goromonzi (*n* = 20) and Mudzi (*n* = 10). Eligible health facilities were those providing and initiating maternal and paediatric ART with at least 30 eligible caregiver-child dyads on their HIV-exposed babies register. Health facilities were selected for the trial based on mapping of all facilities within the district and were at least 15 km apart to minimise the risk of contamination.

### Trial population

The trial population comprised of primary caregivers (biological and non-biological), and their HIV positive or exposed infants aged 0–24 months. Dyads must have been living in the catchment areas of designated trial health care facilities, planning to live full time in the community for the 18 months trial duration, willing to attend meetings in the community once every two weeks, and prepared to provide their residential address and other locator information for follow-ups. Only singleton births were eligible for inclusion. Dyads that included children with chronic illness, not including HIV exposed infants, or children with mental/physical disabilities as recorded by the health worker were excluded.

### Pilot study

A 3-month pilot was conducted prior to the trial (August to October 2015). We recruited 50 caregiver-child dyads seeking health services in five clinics excluded in the trial and geographically separated from the trial clinics. The aim was to assess the acceptability and feasibility of the intervention, to determine feasibility and procedures for trial recruitment, examine preliminary data and to assess the relevance and appropriateness of the parenting programme content adapted to HIV affected families. The pilot resulted in minor changes to the finalised design of the trial and informed the final protocol. These changes included reducing ECS content per module, and increasing modules from 12 to 18, to be conducted fortnightly as opposed to proposed weekly or monthly. Cluster sizes were changed from an initial 16 caregiver-child dyads to either 12 or 24 caregiver-child dyads per cluster to cater for manageable Internal Savings and Lending Scheme (ISALS) composition. These changes informed the final study protocol.

### Trial design

**Fig. 2:**
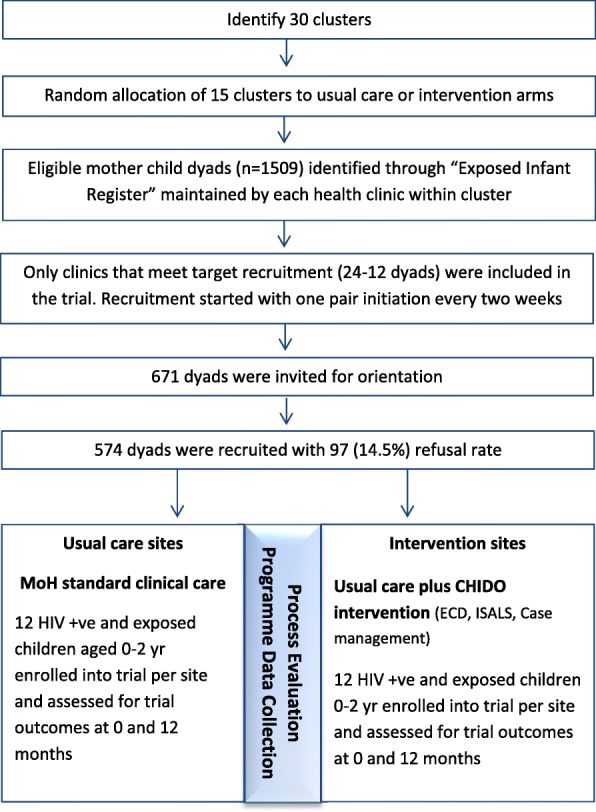
Trial design

Thirty health facilities and their surrounding catchment areas were randomised to the Child Health Intervention for Development Outcomes (CHIDO) intervention or standard of care (Fig. 2). Randomization of the 30 selected clusters was conducted with minimisation on the number of HIV exposed infants (0–24 months) in the Exposed Infant Registers, to ensure that the number of eligible caregiver-child pairs was similar by arm.

### Recruitment and enrolment procedures

A list of eligible infants was extracted from the ‘Exposed Infant Registers’, comprising the name of the biological mother, village, name of the child, date of birth of the child, ART number of the mother and contact details. For the 30 trial clinics there were a total of 1509 eligible infants extracted from the health facility PMTCT registers. Case care workers (CCW) in catchment areas of villages with the highest number of clinics were asked to invite all eligible caregiver-child dyads who met the trial inclusion criteria (*n* = 671) to attend an orientation meeting to learn about the trial. If the biological mother listed in the register was no longer the primary caregiver, the non-biological caregiver was invited to participate as long as the child was listed as HIV exposed. Eligible caregivers who provided verbal consent to participate were booked for enrolment procedures – *n* = 574 and 97 (14.5%) declined enrolment. Enrolment in the CHIDO intervention and usual care arms occurred concurrently, with one clinic from each arm recruited at a time for implementation purposes. Enrolment took 6 months to complete. Follow up for each pair of clinics was 12 months after the CHIDO intervention arm has started receiving the intervention in their community.

All eligible caregiver-child dyads were given trial information and asked to provide written informed consent to participate. Informed consent was collected according to Good Clinical Practice guidelines.

Table 1 summarises the scheduled trial timeline, from participants’ enrolment, intervention roll out, follow up assessments to data analysis phase.

**Table 1:**
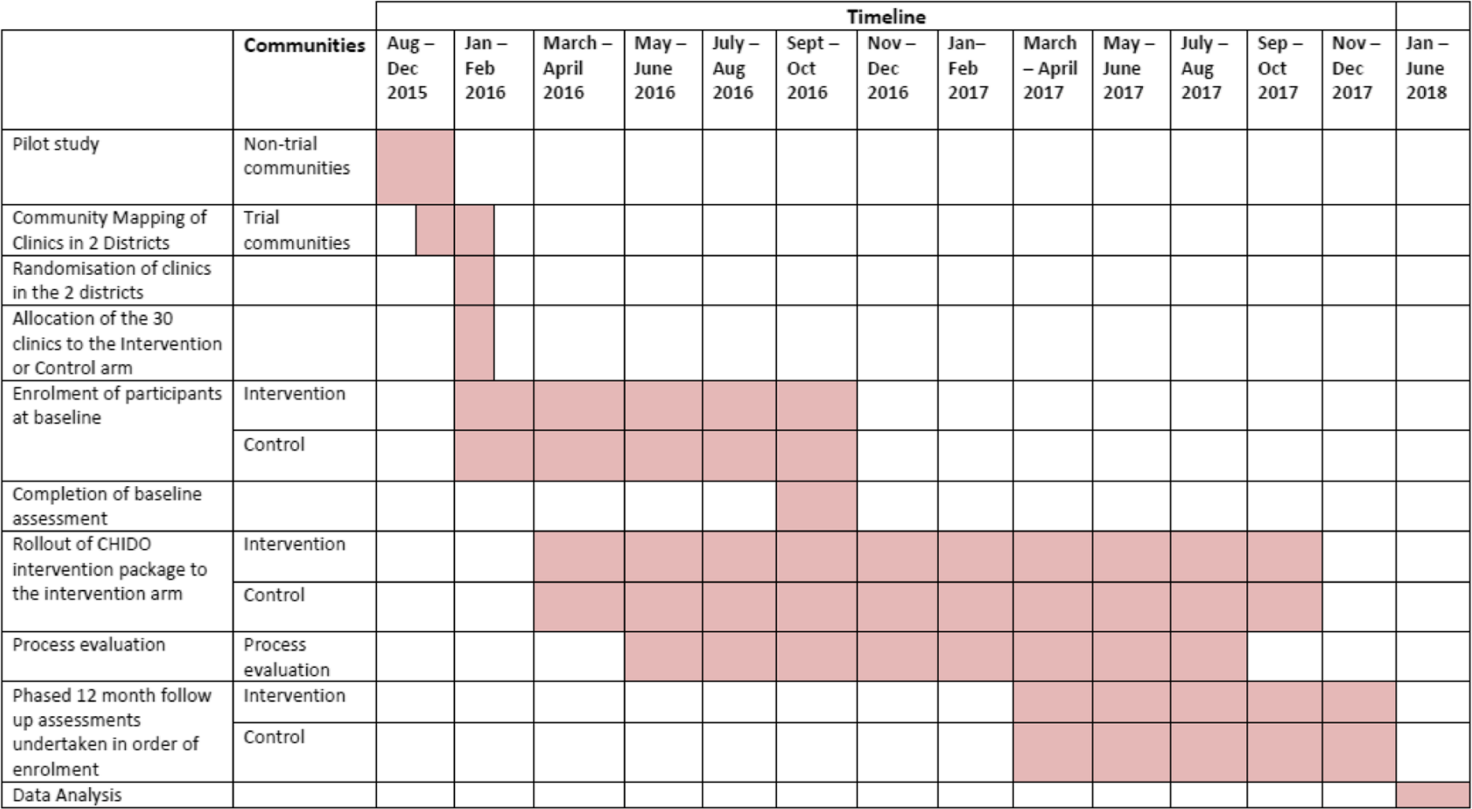
CHIDO trial timeline.

### Intervention components and data collection preparation

#### Usual care arm

Clinics in the usual care arm received the recommended standard of care provided by Zimbabwean Ministry of Health and Child Care (MOHCC) for HIV exposed and/or infected children aged 0–24 month delivered according to 2013 National ART guidelines.

#### Intervention arm

**Fig. 3:**
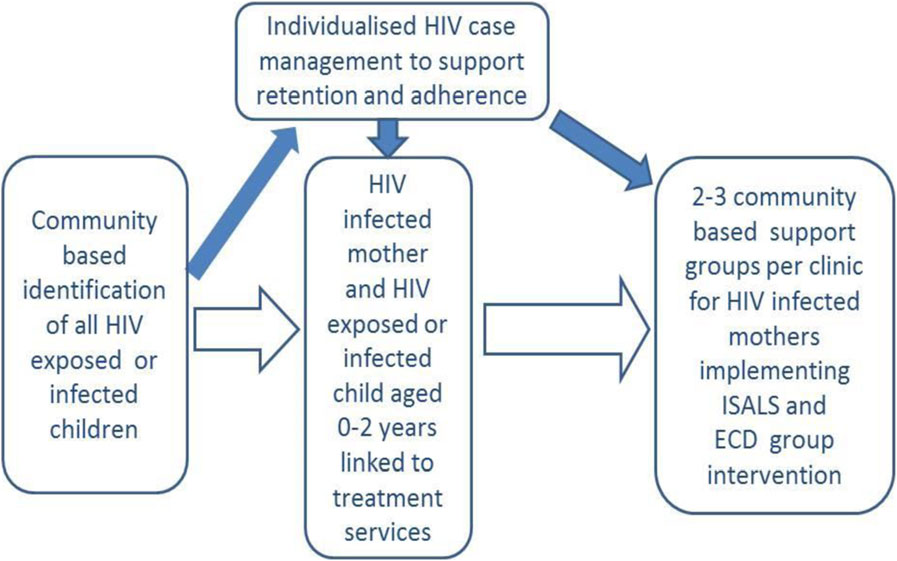
Intervention schema

In addition to usual care, children in the intervention clinics received the CHIDO intervention, a community-based, group intervention, delivered to caregiver-child dyads in groups of 12 every fortnight over 12 months. The intervention had three components i) early childhood stimulation; ii) internal savings and lending scheme to build economic resilience and iii) support from a case care worker to support engagement with and retention in HIV care (Fig. 3). At completion of the trial the details of the intervention will be made available online.

i) **Early childhood stimulation programme;** An evidence-based curriculum to support the promotion of cognitive stimulation, increase use of positive discipline by parents and improve the nutritional status of children was developed and piloted for acceptability and feasibility from existing Zimbabwean (adapted to be age appropriate) and international materials with the aim of strengthening parenting skills. The goals of the curriculum were to a) improve parental promotion of cognitive stimulation; b) improve the nutritional status of children; c) increase the use of positive discipline by parents; and d) enable parents to understand and support the socio-emotional, physical and cognitive development of their children. The ECS curriculum had 18 ninety-minute sessions. A community worker experienced in ECD training, and who received further training and supervision to support caregivers of 0–24 month old facilitated the group. A nurse from the local clinic run some of the medical sessions in the curriculum. The CCW from that community also attended the sessions and assessed the extent to which lessons learnt were then implemented in the home setting when they conduct their home visits. Where mothers needed guidance on implementation of lessons learnt the CCW provided assistance.
ii) **Internal savings and lending scheme;** Caregivers in each ECS groups joined an ISALS group. The main aim was to increase household income to enable them to meet costs such as transport to health facility, clinic user fees, medication and food. These caregivers grouped themselves from within the ECS group into 6–10 members, and agreed to meet fortnightly to save money. Caregivers were trained on how to set up a group, develop a constitution, take meeting minutes and run the ISALS. All groups had different savings options. They could either contribute a set amount to a savings pool at each meeting; or a set amount at the first meeting only; with members able to apply to borrow (and repay) money to cover essential expenditure. If participants were too poor to contribute to an ISALS, they were allowed to earn money through community work to raise their first instalment. Thus those too poor to contribute were enabled to join and save together with those who could afford to join initially.
iii) **Case management;** Caregiver-child dyads recruited to the intervention arm of the trial were supported by CCWs to access paediatric (and adult) HIV services within a continuum of care through the case management system. CCWs within the local communities (also referred to as village health workers) with home-based care experience, received an additional one-week training in ECS and providing adherence support. Home visits by CCWs supported adherence to ART and retention in care, attendance of caregivers and children at fortnightly meetings, as well as monitoring uptake of parenting skills training within the home environment. These activities complemented the monthly review and adherence support provided at the health facility.

In each community, the ECS community worker was responsible for the overall coordination and implementation of the intervention, assisted by the community nurse, ISALS facilitator, and CCW as required. All cadres were expected to attend all intervention group meetings.

### Outcome measures

The primary outcome measures were:

i. **Cognitive development**

The age-standardized Early Learning Composite (ELC) score was assessed for all infants at baseline and endline. Trained assessors (independent of the implementers) used the Mullen Scales of Early Learning focusing on the four cognitive scales (visual reception, fine motor, receptive language, and expressive language). The Mullen Scales of Early Learning Tool is an individually administered comprehensive measure of cognitive functioning for infants and preschool children from birth through 68 months [34]. Scores were adjusted based on the child’s age. The number of assessors was kept to a minimum of 3, to maximise reliability of measurement. To increase validity, assessors were interchanged between intervention and control groups after each clinic pair, to ensure that there was no systematic bias between the groups. The Mullen assessments were video recorded. As quality control, a random 10% sample of videos was reviewed internally and another random 10% sample was reviewed by an independent external assessor prior to the follow up survey.

ii. **Retention in HIV care**

The proportion of HIV exposed or infected children with full retention in care (> 80%) of scheduled visits at 12 months.

Secondary outcomes (Table 2)include HIV infected infants viral load measurement (> 1000 copies per ml at 12 months), ART adherence and retention in both HIV positive mothers and HIV infected infants, parental stress levels and mental health status of caregivers, household food security status and infant nutritional status.

**Table 2:** CHIDO trial outcomes

	Outcome Measures	Target Group	Instrument	Administration
Primary	Child Development Outcomes: Mean childhood development global score	Infants	Mullen Scales for Early Learning	Child Assessment
	Child HIV Outcomes: i) Retention in care	Infants	Questionnaire	Self-report
Secondary	Child HIV Outcomes: i) Viral load	HIV + infants	Viral Load Tests	Clinical/Laboratory Tests
	Child Development Outcomes: i) Visual reception ii) Fine Motor iii) Receptive language iv) Expressive language	Infants	Mullen Scales for Early Learning	Child Assessment
	Nutritional Outcomes: Weight for age, height for age, weight for height (BMI) z-scores	Infants	Mid Upper Arm Circumference tape measure, height mate/board	Child Assessment
	Parenting Outcomes: Parenting Stress Index	Caregivers	Parental Stress Index Short Form (PSI-SF)	Interview
	Adherence Outcomes: i) Retention in care ii) Viral Load	HIV + ve mothers HIV + ve mothers	Medical Adherence Rating Scale (MARS) Viral Load Tests	Interview Clinical/Laboratory Tests
	Food Security Outcome	Caregivers	Household hunger (food deprivation) scale	Interview
	Mental Health Outcomes: i) Postnatal Depression ii) Common Mental Disorders	HIV + ve mothers Caregivers	Edinburgh Postnatal Depression Scale Shona Symptom Questionnaire (SSQ-8)	Interview Interview

### Sample size

Based on data collected in our pilot study, the mean Mullen ELC Score was 110.6 (standard deviation [SD] 16.1). The coefficient of variation (k) between clusters was 0.07 but this does not take account of the clustering effect of the group-based intervention so a value for k of 0.15 was used. Using these values, assuming a harmonic mean of 16 dyads enrolled per cluster and a loss to follow-up of 20%, 15 clusters per arm were needed to provide 80% power to detect an effect size (difference in means/SD) of 1.23 for the Mullen ELC Score. The same sample size provides 80% power to detect a risk difference of 20% in retention in care, assuming retention is 65% in the control arm and k = 0.2. Based on these calculations our recruitment target was 528 caregiver-child dyads in total from 30 clinics. We recruited 574 dyads to ensure a harmonic mean of 24 dyads was enrolled from the larger seven clinics and a harmonic mean of 12 dyads was enrolled from the smaller eight clinics. This was done to ensure ECS group sizes were sufficient to include 2 ISALS groups per ECS group.

### Randomisation process

The imbalance between arms was minimised by using restricted randomization, minimising on the number of HIV exposed infants between 0 and 24 months per clinic. Mapping of all clinic clusters within the two districts was conducted before recruitment and enrolment in each district. Of all the mapped clinics, those with fewer than 30 annual deliveries were excluded from the randomization process. The remaining 30 clusters were randomly allocated to the intervention versus usual care arms. The intervention was delivered to groups of 12 dyads, and 6 health facilities of sufficient size to run one group and 9 facilities of sufficient size to run two groups were allocated to each arm (Fig. 2). To maximise transparency and buy-in from key stakeholders, a public randomisation procedure was undertaken (in January 2016 in the first district and in May 2016 in the second district) involving MOHCC, and district level governance and medical representatives.

### Blinding

Blinding of patients and programme implementers were not possible at baseline because the participants had to know whether they were receiving the ECS programme. At 12 months follow up, the assessments was conducted outside trial communities with participants, from control and intervention communities within the same district, seen together. The assessor was blind to community allocation. Otherwise, data collection procedures were identical.

### Data collection

During the enrolment visit, all participating caregiver-child dyads were allocated a unique identifier to ensure patient confidentiality. Baseline data were collected using an interviewer-administered questionnaire and Audio Computer Assisted Survey Instrument (ACASI) for the sensitive parts of the questionnaire administered with the caregiver and a developmental assessment of the child. Caregivers received guidance on using ACASI from trained surveyors with regards to using the laptop and headphones to listen to instructions, questions, and responses that have been digitally recorded onto the ACASI platform.

The questionnaires collected information on the following domains: demographic characteristics, household characteristics, income and expenditure, food security, antenatal, delivery and post-natal care related to birth of participating child, infant and child feeding practices, maternal mental health, parental stress, HIV testing, disclosure and treatment history for self and participating child, self-reported ART adherence. Questionnaire data were entered directly onto tablets pre-programmed using Open Data Kit with range and consistency checks incorporated.

A follow up assessment was conducted among enrolled caregiver-child dyads after 12 months of programme implementation. This assessment was conducted in the same order in which the clusters were enrolled. Caregivers completed the follow up questionnaire administered in the same way as at baseline. Children had a full developmental assessment conducted by a trained assessor.

### Data management

CeSHHAR Zimbabwe was the data-coordinating centre. In the field, data were uploaded to cloud storage daily and on password-protected office servers at the end of each week. The server was only accessible to the project data manager and named study personnel, on a central computer. Other hard-copy data were stored separately and securely in the field and then locked in a secure room at the data management centre. All data were cleaned, entered, analysed and stored.

### Data analysis

Given the small number of clusters in the trial, cluster-level summary methods will be used. Analysis will be intention-to-treat. Age standardised ELC scores will be calculated from a combination of scores in the four cognitive domains of the Mullen Scales at endline using a HIV negative population in this setting, or a USA reference population if a local reference population cannot be obtained. The mean age-standardised composite score for each cluster will be estimated and the difference in means between arms calculated. The 95% CI for the mean difference will be estimated using a stratified t-test, with variance estimated from the residual mean square from an analysis of variance of cluster-specific means on stratum and trial arm. Analysis will adjust as fixed effects for baseline ELC score, stratum, and the following factors if they are imbalanced between arms at enrolment: age of children and caregivers, HIV status of children, socio-economic status and caregivers’ mental health. For the HIV-specific primary outcomes (proportion of children retained in care at 12 months and proportion with unsuppressed viral load) and for binary secondary outcomes, the unadjusted prevalence ratio will be estimated as the ratio of the geometric mean percentage in the intervention cluster versus the control cluster and compared using logistic regression. The main analysis will be complete case, with multiple imputation as a sensitivity analysis for the two primary outcomes, using variables associated with the outcome and with missingness. Further sensitivity analysis will use individual-level logistic regression and compare the result with the cluster-level methods described above.

### Data sharing

The trial steering committee (principal investigators and investigators) will consider data sharing applications for quantitative and qualitative data. All applications for data sharing must be made through the principal investigator using a standard Data Sharing Form. The trial steering committee convenes a meeting to consider the application. There will be a good reason for turning down a request. Requests will be considered within 4 weeks of being made, and a decision communicated to the applicant as soon as possible thereafter.

### Process evaluation

The process evaluation integrates quantitative and qualitative data collection tools. In addition, key contextual factors that might affect whether the intervention will work in other settings in Zimbabwe were documented. Project documentation will be reviewed to assess whether activities were conducted as scheduled, to identify any delays or gaps, and to check for standardised comparison across intervention sites. Routine programme data were used to track participation in the intervention, complemented by qualitative research, using semi-structured in-depth interviews (IDIs) with ECS staff, case managers, clinic staff and participating caregivers conducted at 3, 6 and 12 months after the start of the programme. There were 24 CCWs, 15 community based trainers (trainers for the ISALS), 17 ECS facilitators, and 13 nurses involved in implementing the intervention.

The research team also conducted an in-depth process evaluation of the ISALS/ECS and case management programme. The process evaluation included gathering general information on programme implementation processes in intervention communities. Participant groups were randomly observed during ISALS collection / ECS sessions to provide data on programme fidelity. Project staff carry an “events diary” to make note of events that were beyond the control of the project, yet have the potential to influence implementation or outcomes (natural disasters, introduction of other ISALS/ECS services, and change in MOHCC standard of care).

IDIs with caregivers were conducted in the intervention arms at two intervals across the project (mid-implementation and end). Caregivers for the IDIs were purposively selected for specific attributes such as high or low levels of engagement in activities, users and non-users of clinical service. These interviews explored perceptions of the community programme, perceptions of clinical and other available services, and positive and negative experiences of the intervention. Semi-structured interviews, with health care staff at intervention clinics and the implementing stakeholders, were conducted to elicit perceptions of the acceptability of the intervention and their own levels of satisfaction and perspectives on its quality.

### Research ethics and approval

Ethical approval has been granted by the Medical Research Council of Zimbabwe (MRCZ/A/1943), Research Council of Zimbabwe, University College London (6789/002) and London School of Hygiene and Tropical Medicine (9912). The exposed Infant register was utilised within the study. This is not a research tool. The register is part of programme data under the Zimbabwe MOHCC data provision. The data are not publicly available and any requests to use that data are made directly to the Zimbabwe MOHCC services and considered on a case-by-case basis.

### Protocol modification

Any deviation from, or changes of, the protocol will be communicated to relevant partners and funding bodies via email. Changes to the current protocol at the Pan-African Clinical Trials Registry will be updated online.

## Discussion

Several studies have highlighted the effectiveness of implementing comprehensive programmes to improve ECD and nutrition outcomes [16, 35]. Research shows poorer child development outcomes in children exposed to HIV especially in resource poor settings [9, 12]. This trial aims to examine the real world effectiveness of a combined parenting and income-generating programme, with the aim of enhancing child stimulation and utilising skill based learning for economic strengthening of populations in resource-limited settings. Prior to the roll out of the trial, the intervention and research procedures were piloted to assess feasibility and acceptability and to inform the details of the final trial design.

Through the 18 ECS sessions, the ISALS training and the home visits over one year we anticipate positive development in growth and cognitive development. We also anticipate improved parental skills, caregiver-child relationships, reduced stress, improved socio-economic resilience and household food security. This trial will contribute towards understanding the effects of integrating different components of care into a comprehensive programme. It will provide useful insight and understanding in delivery methods of comprehensive services and child development in a resource-limited country.

We have used a cluster randomised design to evaluate this community-based intervention as the intervention was delivered at the level of the health facility and it would not have been practical or desirable to randomise individuals within a facility to different standards of care. The trial will determine whether the comprehensive intervention package has an impact on trial outcomes. The detailed process evaluation will allow us to determine whether the intervention was delivered as intended and will provide insights into the strengths and limitations of the different intervention components. It will, however, not be possible to disentangle the effectiveness of the individual intervention components per se. Nevertheless, findings from this trial will contribute to the literature on the effectiveness of parenting interventions and provide insight on how these interventions could be harnessed into the HIV response to maximise child outcome in resource-limited settings.

### Trial status

At the time of the manuscript submission, recruitment for the trial was completed and process evaluation and data analysis ongoing.

## Abbreviations

ACASI: Audio Computer Assisted Self Interviews
ART: Antiretroviral Therapy
CCW: Case Care Workers
CeSHHAR: Centre for Sexual Health and HIV/AIDS Research
CHIDO: Child Health Intervention for Development Outcomes
ECD: Early Childhood Development
ECS: Early Childhood Stimulation
ELC: Early Learning Composite
IDIs: In-depth Interviews
ISALS: Internal Savings and Lending Scheme
MOHCC: Ministry of Health and Child Care
SD: Standard Deviation
